# Interrupted-time-series analysis of the immediate impact of COVID-19 mitigation measures on preterm birth in China

**DOI:** 10.1038/s41467-022-32814-y

**Published:** 2022-09-03

**Authors:** Yanxia Xie, Yi Mu, Peiran Chen, Zheng Liu, Yanping Wang, Qi Li, Mingrong Li, Juan Liang, Jun Zhu

**Affiliations:** 1 National Office for Maternal and Child Health Surveillance of China, West China Second University Hospital, Sichuan University, Chengdu, Sichuan China; 2 Department of Paediatrics, West China Second University Hospital, Sichuan University, Chengdu, Sichuan China; 3 Department of Obstetrics, West China Second University Hospital, Sichuan University, Chengdu, Sichuan China; 4 Key Laboratory of Birth Defects and Related Diseases of Women and Children (Sichuan University), Ministry of Education, Chengdu, Sichuan China

**Keywords:** SARS-CoV-2, Epidemiology, Neonatology

## Abstract

Preliminary evidence from China and other countries has suggested that coronavirus disease 2019 (COVID-19) mitigation measures have caused a decline in preterm births, but evidence is conflicting. Utilising a national representative data of 11,714,947 pregnant women in China, we explored the immediate changes in preterm birth rates during the COVID-19 mitigation period using an interrupted-time-series analysis. We defined the period prior to February 1, 2020 as the baseline, followed by the COVID-19 mitigation stage. In the first month of the COVID-19 mitigation, a significant absolute decrease in preterm birth rates of 0.68% (95%CI:−1.10% to −0.26%) in singleton, and of 2.80% (95%CI:−4.51% to −1.09%) in multiple births was noted. This immediate decline in Wuhan was greater than that at the national level among singleton births [−2.21% (95%CI:−4.09% to −0.34% vs. −0.68%)]. Here we report an immediate impact of COVID-19 mitigation measures on preterm birth in China.

## Abstract (web summary)

The broader impacts of COVID-19 mitigation measures may include changes in the incidence of preterm birth. Here, the authors use data from ~11.7 million pregnancies in China and find evidence of a small decline in preterm birth rates immediately following the implementation of COVID-19 mitigation measures.

## Introduction

Preterm birth is defined as any birth prior to 37 completed weeks of gestation, or fewer than 259 days from the first day of a woman’s last menstrual period, according to the World Health Organization^1,2^. Such deliveries occur in an estimated 10.6% of pregnancies globally, and the rate has increased over time^3^. The same trend is seen in China (from 5.9% in 2012 to 6.4% in 2018), especially after the universal two-child policy took effect^4^. Preterm births place an enormous burden on the families of preterm newborns with regard to their offspring’s mortality and morbidity^3,5–7^. Families of preterm newborns often experience considerable psychological and financial hardship^5,7^. Regarding the infant, complications of preterm birth was the leading cause of death in children younger than five years of age in 2016, accounting for approximately 16% of all deaths, and the leading cause of death (35%) among neonates (i.e., in the first 28 days of life) globally^3^. In addition, preterm birth accounts for 75% of perinatal mortality and more than half of long-term childhood morbidity^6^. Therefore, reducing the incidence of preterm birth is a global priority for infant health.

Currently, primary and secondary preventive strategies for preterm birth are deficient owing to a lack of understanding of its pathophysiology^8^. However, its risk factors have been studied extensively, and the known factors can be roughly divided into non-modifiable and modifiable risk factors. Modifiable risk factors include infection, work-related stress, exposure to certain environmental pollutants, and a series of lifestyle factors^9^. In 2015, the United Nations Sustainable Development Goal 3 (SDG 3) proposed the elimination of preventable deaths of newborns and children aged under five years following the global failure to achieve Millennium Development Goal 4. Achieving SDG 3 targets at an earlier date than planned requires more attention to modifiable risk factors of preterm birth and greater efforts to explore strategies to prevent preterm birth. Recently, numerous studies conducted in multiple countries assessed the effect of coronavirus disease 2019 (COVID-19) lockdown measures on the incidence of preterm birth and obtained remarkably varied results^10–20^. Several studies from the Netherlands, Denmark, Ireland, Italy, and Japan reporting on reduction in preterm birth following the implementation of lockdown measures has raised hopes for effective preterm birth prevention in the future; however, these studies need more evidence-based support^11,13,19,20^. The link between preterm birth rate changes and COVID-19 mitigation measures have been identified in two studies carried out in China; however, the conclusions from these studies were contradictory^21,22^. Both studies were single-centre studies and have relatively small sample sizes. In addition, all studies were restricted to singleton births. Moreover, few studies have assessed the differential impact of lockdown measures on iatrogenic and spontaneous preterm deliveries or across the socioeconomic status (SES) strata^10–13,19^ due to a lack of case-specific demographic data. An assessment of this association has also not been conducted in the whole of China, one of the world’s most populous, diverse countries, and one that has implemented some of the strictest COVID-19 mitigation measures and best outbreak control strategies^23^. China’s National Maternal Near Miss Surveillance System (NMNMSS) provides an opportunity for further research. NMNMSS, established in October 2010 by the National Health Commission of China, was designed to monitor women’s health status based on case information obtained from 438 member hospitals across 326 urban districts and rural counties in 30 provinces, and represented 8‒10% of all births across China.

In this work, we aimed to use data from the NMNMSS to assess the immediate changes in preterm birth rate during the COVID-19 mitigation period in China, including changes related to the various categories of births/pregnancies along with concomitant changes in stillbirth rates. Furthermore, we also explored whether the impacts on preterm births associated with mitigation measures varied by SES.

## Results

### The study population and its representativeness

During the study period (January 1, 2012 to December 31, 2020), 12,294,471 women delivered at least one baby who was more than or equal to 28 weeks of gestation or weighed 1000 g or more. After excluding pregnancies lacking data, 11,714,947 women were included in the final analysis (11,504,271 delivered singletons and 210,676 delivered multiples). A more significant proportion of births were monitored by NMNMSS in 2020 (9.44%) than in previous years, and the proportion of births captured in February 2020 (8.98%) and March 2020 (9.47%), using strict epidemic mitigation measures, was also higher than those in previous years. (Supplementary Fig. 1, Supplementary Fig. 2).

Included in the analysis were 108 months of preterm birth rates. Of these, 97 months were from baseline (January 1, 2012 to January 31, 2020) and 11 months were from the intervention stage (February 1, 2020 to December 31, 2020).

### Trends in maternal characteristics

The sociodemographic characteristics of the women who gave birth before and after the implementation of COVID-19 mitigation measures are presented in Table 1. During the study period, the proportion of singleton births among mothers with an advanced age (≥35 years) gradually increased from 9.1% to 12.0%, while multiple births increased from 11.3% to 13.8%. Moreover, the proportion of singleton and multiple pregnancies in the advantaged population increased slightly. In contrast, maternal characteristics were notably the similar in the months before (nine months immediately prior to the intervention) and after COVID-19 mitigation measures were implemented.

**Table 1:** Maternal sociodem graphic characteristics by intervention in China, 2012–2020 (*N* = 11,714,947)

		Singleton births characteristics, *n* (%)			Multiple births characteristics, *n* (%)		
		Baseline		Intervention stage	Baseline		Intervention stage
		2012m1 –2020m1	2019m5–2020m1		2012m1 –2020m1	2019m5–2020m1	
**Total,** ***n***	10,502,357		1,015,359	1,001,914	191,594	19,653	19,082
**Maternal age (y)**							
Mean (SD)	28.40 ± 4.97		29.67 ± 4.70	29.91 ± 4.76	29.53 ± 4.86	30.62 ± 4.57	30.84 ± 4.51
<35	9,546,633(90.90%)		900,661(88.70%)	881,880(88.02%)	169989(88.72%)	16,990(86.45%)	16,443(86.17%)
>=35	955724(9.10%)		114698(11.30%)	120,034(11.98%)	21,605(11.28%)	2,663(13.55%)	2,639(13.83%)
**Social status**							
Advantaged	6,286,513(59.86%)		719,375(70.85%)	703,031(70.17%)	120302(62.79%)	13,749(69.96%)	13077(68.53%)
Disadvantaged	4,215,844(40.14%)		295,984(29.15%)	298,883(29.83%)	71292(37.21)	5,904(30.04%)	6005(31.47%)
**Location**							
Rural	4,286,165(40.81%)		337,277(33.22%)	350,565(34.90%)	43,806(22.86%)	3,367(17.13%)	3,563(18.67%)
City	6,216,192(59.19%)		678,082(66.78%)	651,348(65.01%)	147,788(77.14%)	16,286(82.87%)	15,519(81.335)
**Parity**							
Nulliparas	5,901,659(55.24%)		507,013(49.93%)	488,028(48.71%)	121,454(63.39%)	12,508(63.64%)	12,040(63.10%)
Multiparas	4,700,698(44.76%)		508,346(50.07%)	513,886(51.29)	70,140(36.61%)	7,145(36.36%)	7,042(36.90%)
**Stillbirth**	74,703(0.71%)		5,454(0.54%)	5,689(0.57%)	2,454(1.28%)	171(0.87%)	193(1.01%)
**Preterm**	619,479(5.94%)		61,786(6.12%)	64,357(6.46%)	98,737(52.21%)	10,666(54.75%)	10,676(56.52%)
Very preterm	68,054(0.65%)		7,116(0.70%)	7,460(0.75%)	10,411(5.51%)	1,156(5.93%)	1,155(6.11%)
Moderate preterm	83,690(0.80%)		8,294(0.82%)	8,383(0.84%)	14,850(7.85%)	1,596(8.19%)	1,506(7.97%)
Late preterm	467,735(4.49%)		46,376(4.59%)	48,514(4.87%)	73,476(38.85%)	7914(40.63%)	8,015(42.43%)
**Term**	9,702,936(93.06%)		944,045(93.48%)	928,581(93.21%)	89,204(47.17%)	8721(44.77%)	8,135(43.07%)
**Post term**	99,357(0.95%)		3,317(0.33%)	2,533(0.25%)	489(0.26%)	18(0.09%)	13(0.07%)
**Spontaneous preterm**	346,764(3.33%)		31,966(3.17%)	32,753(3.29%)	29,207(15.44%)	2,754(14.14%)	2,730(14.45%)
**Iatrogenic preterm**	272,715(2.62%)		29,820(2.95%)	31,604(3.17%)	69,530(36.77%)	7,912(40.62%)	7,946(42.07%)

*SD* standard deviation.

Baseline: January 1, 2012 to January 31, 2020.

Intervention stage: February 1, 2020 to December 31, 2020.

### Changes in the preterm birth rate due to COVID-19 mitigation measures

In both singleton and multiple births, linear trends in preterm birth rate (singletons: *p* < 0.001; multiple: *p* < 0.001) and stillbirth rate (singletons: *p* < 0.001; multiple: *p* < 0.001) were observed from 2012 to 2020, using the Cochran Armitage test.

**Fig. 1:**
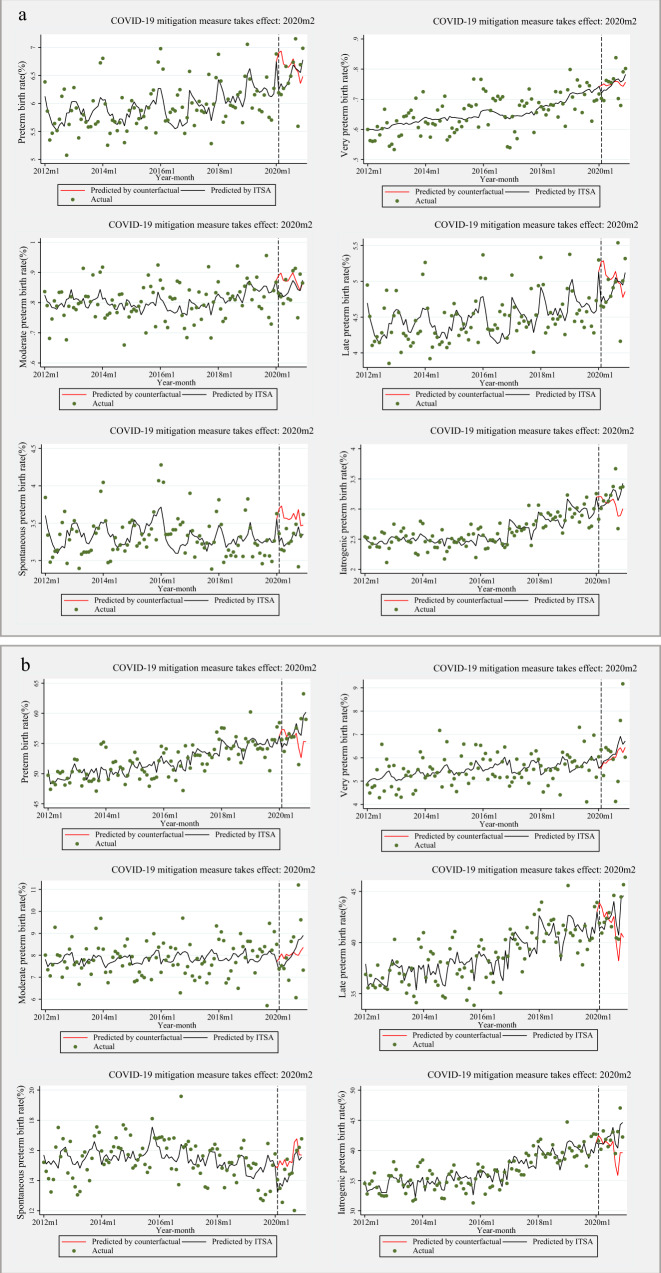
Interrupted time series analysis (ITSA) of the preterm birth rate across different type pregnancies in China, 2012–2020. **a** singleton births; **b** multiple births. Dots indicate true monthly preterm birth rates, solid lines indicate the mean of estimated preterm birth rates per month by ITSA model, and red solid lines indicate the mean of estimated preterm birth rates per month assuming that COVID-19 mitigation measure had not occurred by ordinary least-squares regression with Newey-West standard errors. Baseline: from January 1, 2012 to January 31, 2020; Intervention stage: from February 1, 2020 to December 31, 2020. COVID-19: coronavirus disease 2019. The *p* values are two-sided.

Table 2 and Fig. 1 presents the interrupted time series analysis (ITSA) results for preterm singleton and multiple births. As shown, the intercept of the singleton preterm birth rate was estimated at 8.91%, and the rate appeared to significantly increase every month before intervention (2020m2) by 0.03% (95% confidence interval [CI] 0.01% to 0.05%, *p* = 0.002). In the first month of the intervention, there was a significant immediate absolute decrease in the singleton preterm birth rate by 0.68% (95% CI−1.09% to −0.26%, *p* = 0.002), followed by a significant increase in the rate (relative to the pre-intervention trend) of 0.10% per month (95% CI 0.05% to 0.14%, *p* < 0.001). The immediate decline following the COVID-19 mitigation measures was mainly due to a decrease in moderate and late preterm births (Table 1**;** Fig. 1a). The intercept of preterm birth rate for multiple births was eatimated at 50.56%, and the rate increased every month prior to intervention by 0.06% (95% CI 0.001% to 0.11%, *p* = 0.048); however, an immediate absolute reduction of 2.80% (95% CI − 4.51% to −1.09%, *p* = 0.002) was subsequently observed in the first month of the COVID-19 mitigation measures, followed by a significant rate increase of 0.80% (95% CI: 0.38% to 1.22%, *p* < 0.001) per month (relative to the pre-intervention trend). The immediate decline following the COVID-19 mitigation measures was mainly attributed to late preterm births (Table 1**;** Fig. 1b). In either singleton or multiple births, the immediate absolute reduction was not observed after a two-month lag following the implementation of COVID-19 mitigation measures (Supplementary Table 1, Supplementary Fig. 3).

**Table 2:** Interrupted time series analysis of monthly preterm birth rate in singleton and multiple pregnancies in China, 2012–2020

	Singleton births			Multiple births		
	β (Coefficient)	*P*-value	95%CI	β (Coefficient)	*P*-value	95%CI
**Preterm**						
Baseline intercept	8.91	<0.001	6.02–11.81	50.56	<0.001	39.75–61.36
Baseline slope	0.030	0.002	0.011–0.049	0.056	0.048	0.001–0.112
Intervention-Intercept change	−0.675	0.002	−1.091~−0.260	−2.797	0.002	−4.507~−1.088
Intervention-Slope change	0.095	<0.001	0.048–0.142	0.801	<0.001	0.384–1.218
**Very preterm**						
Baseline intercept	0.77	<0.001	0.43–1.12	4.35	0.011	1.00–7.71
Baseline slope	0.003	0.052	−0.001–0.005	0.009	0.350	−0.011–0.029
Intervention-Intercept change	-0.025	0.375	−0.082–0.031	0.186	0.715	−0.819–1.191
Intervention-Slope change	0.005	0.215	−0.003–0.013	0.026	0.843	−0.235–0.288
**Moderate preterm**						
Baseline intercept	1.06	<0.001	0.653–1.468	6.15	0.026	0.75–11.55
Baseline slope	0.003	0.067	−0.001–0.005	−0.001	0.927	−0.027–0.024
Intervention-Intercept change	-0.073	0.005	−0.123~−0.022	−0.424	0.310	−1.248–0.400
Intervention-Slope change	0.008	0.004	0.003–0.013	0.113	0.090	−0.018–0.244
**Late preterm**						
Baseline intercept	7.08	<0.001	4.52–9.64	40.06	<0.001	31.35–48.76
Baseline slope	0.025	0.002	0.009~0.041	0.048	0.046	0.001–0.095
Intervention-Intercept change	−0.577	0.001	−0.921~−0.234	−2.559	<0.001	−3.812~−1.307
Intervention-Slope change	0.082	<0.001	0.050–0.114	0.662	<0.001	0.481–0.843
**Spontaneous preterm**						
Baseline intercept	5.41	<0.001	3.43–7.39	19.70	<0.001	13.06–26.35
Baseline slope	0.020	0.007	0.005–0.034	0.036	0.082	−0.005–0.077
Intervention-Intercept change	-0.458	0.005	−0.773~−0.142	−1.714	0.015	−3.093~−0.334
Intervention-Slope change	0.032	0.143	−0.011–0.075	0.153	0.242	−0.105–0.412
**Iatrogenic preterm**						
Baseline intercept	3.50	<0.001	2.52–4.49	30.85	<0.001	22.07–39.63
Baseline slope	0.011	<0.001	0.005–0.016	0.020	0.413	−0.028–0.068
Intervention-Intercept change	−0.218	0.019	−0.399~−0.036	−1.084	0.208	−2.779~–0.612
Intervention-Slope change	0.063	<0.001	0.036~0.090	0.648	<0.001	0.303–0.992

The *p* values are two-sided.

CI: confidence interval.

Baseline: January 1, 2012 to January 31, 2020.

Intervention stage: February 1, 2020 to December 31, 2020.

When stratified into spontaneous and iatrogenic preterm births, the immediate decline of spontaneous preterm births after intervention was observed among singleton (Table 1, Fig. 1a) and multiple pregnancies (Table 1, Fig. 1b). In terms of iatrogenic preterm births, the immediate decrease was only observed among singleton pregnancies (Table 1, Fig. 1a, Fig. 1b). Compared to iatrogenic preterm births, spontaneous preterm births decreased more after implementation of strict COVID-19 mitigation measures among singleton pregnancies (−0.46% vs. −0.22%). Furthermore, no immediate increase in stillbirth rates were observed along with the implementation of COVID-19 mitigation measures (Supplementary Fig. 4). A similar pattern was observed when assessing 2016–2020 data as opposed to 2012–2020 data (Supplementary Table 2).

### Heterogeneity in effects across pregnancies of different SESs

**Fig. 2:**
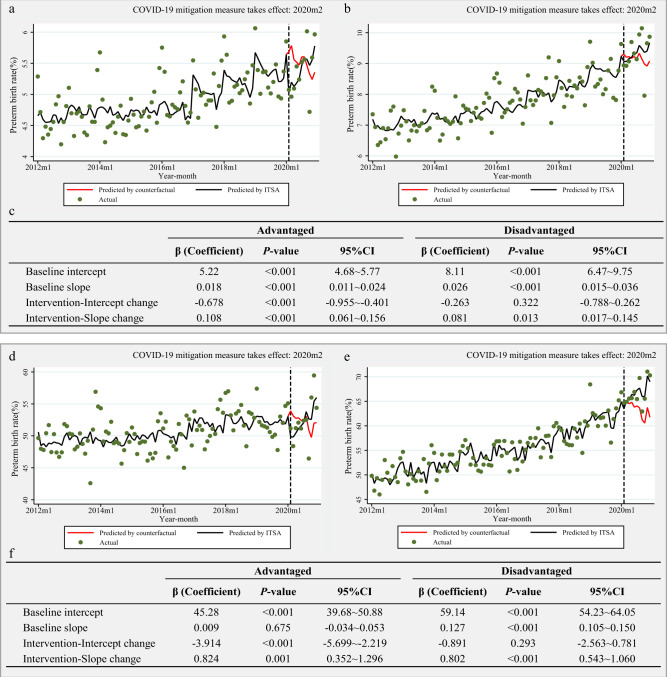
Interrupted time series analysis (ITSA) of the preterm birth rate across different socioeconomic statuses in China, 2012–2020. **a** advantaged in singleton births; **b** disadvantaged in singleton births; **c** the result of ITSA in singleton births across population; **d** advantaged in mutiple births; **e** disadvantaged in mutiple births; **f** the result of ITSA in multiple births across population. Dots indicate true monthly preterm birth rates and solid lines indicate the mean of estimated preterm birth rates per month by ITSA model, and red solid lines indicate the mean of estimated preterm birth rates per month assuming that COVID-19 mitigation measure had not occurred by ordinary least-squares regression with Newey-West standard errors. Women who were illiterate, or had only primary school education, who were unmarried, or who had fewer than five antenatal visits were defined as the disadvantaged group. On the contrary, women with middle school or above education, married, and had more than five antenatal visits were defined as the advantaged group. Baseline: from January 1, 2012 to January 31, 2020; Intervention stage: from February 1, 2020 to December 31, 2020. Intercept change: change in level compared with the previous stage; Slope change: change in trend compared with the previous stage, per month. CI confidence interval, COVID-19 Coronavirus disease 2019. The *p* values are two-sided.

We observed heterogeneity in the magnitude of relative changes in preterm births across different SESs (Fig. 2). Regardless of being a singleton or multiple pregnancy, a higher SES (advantaged group) was associated with a significantly greater immediate reduction in the preterm birth rate when the initial COVID-‍19 mitigation measures took effect compared to the preterm birth rate in a lower SES (disadvantaged group).

### Sensitivity analyses

**Fig. 3:**
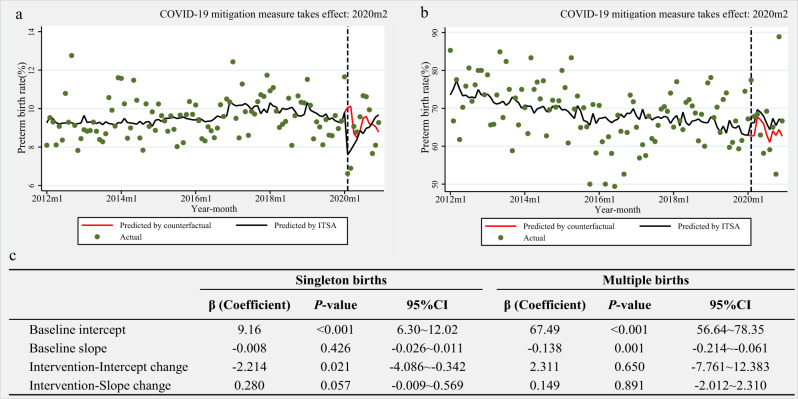
Interrupted time series analysis (ITSA) of the preterm birth rate across different type pregnancies in Wuhan, 2012–2020. **a** singleton births; **b** multiple births; **c** the result of ITSA across different type pregnancies. Dots indicate true monthly preterm birth rates and solid lines indicate the mean of estimated preterm birth rates per month by ITSA model, and red solid lines indicate the mean of estimated preterm birth rates per month assuming that COVID-19 mitigation measure had not occurred by ordinary least-squares regression with Newey-West standard errors. Baseline: from January 1, 2012 to January 31, 2020; Intervention stage: from February 1, 2020 to December 31, 2020. Intercept change: change in level compared with the previous stage; Slope change: change in trend compared with the previous stage, per month. CI: confidence interval; COVID-19: coronavirus disease 2019. The *p* values are two-sided.

We conducted a sensitivity analysis restricted to births in Wuhan (Fig. 3), the area that was most affected by the COVID-19 outbreak. For singleton pregnancies, a stable trend in preterm birth rate was observed from baseline (−0.01% per month, 95% CI − 0.03% to 0.01%, *p* = 0.426), whereas an immediate absolute decline by 2.21% (95% CI−4.09% to −0.34%, *p* = 0.021) was observed after the implementation of COVID-19 mitigation measures, followed by a steady trend of 0.28% per month (95% CI −0.01% to 0.57%, *p* = 0.057) based on the baseline trend. The immediate reduction in the preterm birth rate in Wuhan was greater than that at the national level among singleton births (−2.21% vs. −0.68%). However, the preterm birth rate remained stable after the implementation of COVID-19 mitigation measures among multiple pregnancies in Wuhan (Figs. 3b,c). Similarly, no significant immediate increase in the stillbirth rate was observed along with the implementation of COVID-19 mitigation measures in Wuhan (Supplementary Fig. 5).

Furthermore, Supplementary Fig. 6 depicts the change in preterm birth rate when we assumed December 2019 and January 2020 as the cut-off points of the interrupted time series analysis; however, an immediate decrease in preterm birth rates were not noted during this period.

## Discussion

In the context of the global COVID-19 pandemic, we used national representative cohort data to assess immediate changes in the preterm birth rate after the implementation of lockdown measures in China. The longstanding trend of increasing preterm birth rates in China still continues; however, we found a subtle yet significant immediate decrease in the monthly preterm birth rate for both singleton and multiple pregnancies, and no immediate increase in the stillbirth rate, after the implementation of COVID-19 mitigation measures. These decreases could be attributed to a decrease in moderate and late preterm births among singleton pregnancies, and a decrease in late preterm births among multiple pregnancies. The immediate decline in preterm birth rates in Wuhan was greater than that at the national level. During the period of study, an immediate decline in spontaneous preterm births was observed among singleton and multiple pregnancies, whereas a decline in iatrogenic preterm births was exclusively observed among singleton pregnancies. Among singleton pregnancies, the short-term reduction in spontaneous preterm births was greater than that in iatrogenic preterm births. The COVID-19 pandemic and associated lockdown measures have exacerbated existing health inequalities in preterm birth rates across different SES. Furthermore, this immediate downward in preterm birth rates was not observed after a two-month lag following the implementation of COVID-19 mitigation measures.

To the best of our knowledge, our study is the first to explore the effects of COVID-19 mitigation measures on the preterm birth rate in relevant subgroups by using a large, high-quality national representative database, in China. Moreover, 95.1% of births in each member institution was captured by NMNMSS, which operated steadily during the COVID-19 epidemic period ensuring that the selection bias caused by the epidemic was limited. The COVID-19 mitigation period provided a valuable epidemiological research opportunity to assess the impact of changes in modifiable risk factors on the occurrence of preterm births. To assess its impact, we developed a month-based dataset including singleton and multiple pregnancies, which represented 8–10% of all pregnancies in China, based on a large, nationally representative, and hospital-based registry. We focused on the immediate change in the preterm birth rate during the COVID-19 mitigation period rather than on the rate of preterm birth in China. Previous studies have indicated that they could not discern whether changes in demographic composition of the population following the COVID-19 pandemic might have contributed to the findings (initial implementation of COVID-19 mitigation measures was associated with a substantial reduction in the incidence of preterm births in the following months) as their datasets did not have individual-level information on relevant covariates^19^. Conversely, our dataset can do a better extension to this part of the research, we can precisely observe the influence of COVID-19 mitigation measures on immediate changes of the preterm birth rate after controlling for individual-level information on relevant covariates. Therefore, the 8.91% in our study is the intercept of preterm birth rate estimated by the ITSA model after adjusting for maternal age, parity, education level, and the presence of eclampsia, while the preterm birth rate of 5.9% to 6.4% previously reported did not take these factors into account^4^.

To control the COVID-19 outbreak, China banned travel to and from Wuhan City on 23 January 2020; all provinces, municipalities, and autonomous regions in mainland China subsequently activated a Level 1 response over the following weeks^24,25^. As an emergency response, China implemented a range of behavioural and clinical interventions to mitigate the pandemic in China and worldwide^23,26,27^. Behavioural interventions included the following aspects: (1) the use of personal protective equipment—particularly the wearing of face mask-wearing; (2) regular hand-washing or use of an alcohol-based hand sanitizer; (3) reduction in physical contact advising against handshaking and physical touch; (4) maintaining social distance—advice against social interactions and visits; (5) closing schools, factories, and entertainment venues; (6) banning public gatherings, encouraging working from home, and reducing commuting to and from work/home; and (7) recommendations to stay at home when experiencing symptoms (fever or respiratory complaints), after coming in contact with someone who tested positive for COVID-19, or after having visited a high-risk area. Recently, Deng et al. reported that the preterm birth rate had slightly increased before the universal two-child policy took effect in China, while a steeper increase was observed after implementation of this policy^4^. However, we found a brief reversal in this upward trend, where an immediate decline in the preterm birth rate was observed among singleton and multiple pregnancies following the implementation of COVID-19 mitigation measures. These findings are based on controlling for possible seasonality on preterm birth rates, which are consistent with those of studies from the Netherlands, Denmark, Ireland, Italy, and Japan^11,13,19,20^. However, several studies have raised doubts regarding whether the observed reductions in preterm birth occurred at the expense of an increase in stillbirths^11,12,18,19^. Therefore, we further investigated the immediate fluctuations in stillbirth rates and found no significant changes after the implementation of mitigation measures, confirming that the immediate decline in preterm birth did not occur at the expense of an increase in stillbirths. Perinatal birth in China is defined as the birth of a foetus after 28 completed weeks of gestation, or at a birth weight of 1000 g or heavier, which is much later than that of 20 or 22 weeks used to define the term in most developed countries^28^. We reassessed the impact of COVID-19 mitigation on preterm births and stillbirths according to the definitions of preterm birth and stillbirth in some developed countries (≥22 w), and the results were similar (Supplementary Table 3). Moreover, this decline only occurred during the period of the most stringent mitigation measures. Although scattered cases have been reported in December 2019, there was no downward trend in preterm birth given that no public intervention measures were applied during that period. In addition, no further immediate downward trend of preterm birth rates were observed after a two-month lag when the COVID-19 mitigation measures were implemented. Wuhan, the epicentre of the pandemic, has been affected by harsher lockdown measures than elsewhere, including the entire lockdown of the residential community. The average monthly decline in preterm birth in Wuhan was greater than that at the national level among singleton pregnancies. Notwithstanding the stable preterm birth rates among multiple pregnancies during the study period in the Wuhan population, it may have been influenced by a variety of uncontrolled biases. We speculate that the implementation of strict COVID-19 mitigation measures may be closely associated with the decline in preterm birth, with stricter measures leading to a greater decline.

In our study, the immediate decrease in the preterm birth rate was mainly due to a decrease in moderate and late preterm births among singleton pregnancies, and a decrease in late preterm births among multiple pregnancies. Our findings are consistent with those of a study from the Netherlands, which suggested that the decrease in preterm birth is fairly constant regardless of the gestational age, although the vast majority of preterm infants are born moderate or late preterm (i.e., at 32 weeks and 0 days to 36 weeks and 6 days)^19^. We did not observe a declining trend in very preterm birth rate because it has not been possible to prevent very preterm births, mainly due to the complexity of its causative factors, such as abnormalities of the reproductive system, advanced maternal age, and serious maternal and foetal diseases^29^. Been et al. stressed the importance of exploring the differential impact of COVID-19 mitigation measures on spontaneous and induced preterm deliveries, and the lack of results based on robust statistical methods to confirm whether the lockdown measures made any difference^19^. Our study fills this gap in the literature. We found that spontaneous preterm births accounted for 55.5% of singleton and 29.2% of multiple preterm births. The reduction in spontaneous preterm births among singleton and multiple pregnancies following the implementation of COVID-19 mitigation measures further suggested that spontaneous preterm birth is controllable and that many of the known modifiable risk factors of preterm birth might have been affected by lockdown measure implementation^9,19^. These include asymptomatic maternal infection, which can cause intrauterine infection through vertical transmission. Social distancing, self-isolation, lack of commuting, closing of schools and childcare facilities, and an increased awareness of hygiene (e.g., hand washing) can reduce contact with pathogens and subsequent risk of infection. The parallel and unprecedented reduction in influenza and other respiratory viral infections that was observed in New Zealand during the COVID-19 pandemic serves as strong evidence for this hypothesis^30^. These indicate that hygiene measures and anticipatory behavioural changes may have contributed to the timing of the decrease in the preterm birth rate^19^. In addition, the closure of most businesses and working from home may have led to less physically demanding activities, reduced shift work, less work-related stress, optimisation of sleep duration, uptake of indoor and outdoor maternal exercise, and increased social and family support, all contributing to possible beneficial effects. Furthermore, the decrease in air pollution caused by the mitigation measures may have also played a key role in the observed reduction in premature birth^31^, given that air pollution—particularly anthropogenic PM2.5 which can remain airborne for extended periods and travel hundreds of miles—is associated with 18% of premature births globally^11^.

An immediate decrease in the iatrogenic preterm birth rate was only observed among singleton pregnancies; however, this decrease was lower than that in spontaneous singleton preterm births. It should be noted that the increased preterm birth rate in recent years can be partly attributed to changes in the use of obstetric interventions^32–34^. In the United States, clinicians have been urged to reduce nonmedically indicated or elective deliveries prior to 39 weeks^34^. Hypertensive disorders in pregnancy, placental factors, foetal distress, multiple pregnancies, intrahepatic cholestasis during pregnancy, and pregnancy combined with medical and surgical diseases constitute the main causes of iatrogenic preterm deliveries in China^35^. Obstetricians should have a good understanding of the timing of delivery related to various complications. In cases where termination of pregnancy prior to 37 weeks is contraindicated, premature intervention should be avoided to reduce unnecessary iatrogenic preterm delivery. The observed immediate reduction in the iatrogenic preterm birth rate in singleton pregnancies may be due to the lack of obstetric intervention, in pregnancies that did not require emergency intervention, because of isolation and limited medical resources during the COVID-19 mitigation period. This suggests that some iatrogenic preterm births may be controlled. However, we did not find a similarly decreasing trend in iatrogenic preterm birth rates in multiple pregnancies. Multiple gestation, accounting for only 2–3% of pregnancies, carries a substantial risk of preterm birth, constituting 15–20% of all preterm births^2^. The causes of preterm birth due to a multiple pregnancy are complex and relatively uncontrollable; therefore, studies are often limited to singleton pregnancies. We included multiple pregnancies to explore whether COVID-19 mitigation measures have a beneficial effect on the preterm birth rate in these cases, despite their inherent increased risk of preterm delivery.

The change in preterm birth rates during the lockdown period differed between populations with different SESs. An immediate reduction in preterm birth rates was exclusively found in the advantaged group with a high SES. Compared with women with low SESs, those with high SESs have a stronger sense of self-preservation due to their advanced educational levels, social status, and stable financial income. This renders them more likely to obtain protective products, in addition to having more purchase channels and the financial capacity to acquire them^36^, while women with low SESs may face more financial pressure, forcing them to remain in contact with the outside world, which may increase their chances of infection leading to preterm birth. The pandemic and associated lockdown measures may have aggravated the existing health and socioeconomic inequalities within populations^37–39^. Nevertheless, studies from California and Philadelphia in the United States reported no significant change in the rate of preterm birth after lockdown^15,17^. The differences between our findings and those of the above-mentioned studies could be explained by the fact that multiple risk factors for preterm delivery that might be responsive to lockdown measures vary across populations and that mitigation measures may have been inadequately implemented. The decline in preterm births may have been even greater during the mitigation period if the population with a low SES behaved similarly to the population with a high SES. Therefore, there is an urgent need to prioritise accessible and equitable health services as a strategic response to this pandemic and future health crises.

One major limitation of our retrospective study was the presence of residual confounding factors, especially in the analysis of multiple pregnancies. Some important risk factors, such as the use of assisted reproductive technology and foetal complications, were not included in the NMNMSS. Future analyses should consider these covariates. Another limitation was that we calculated the gestational age using the last menstrual period when early pregnancy ultrasound is now considered a gold standard. Previous studies have shown that use of the last menstrual date may overestimate gestational age by approximately 2 days (range: 0.2‒2.8 days) compared with ultrasound, but the sensitivity and positive prediction of the last menstrual date in identifying preterm birth value is relatively high^40–42^. Therefore, the effect of underestimation is assumed to be relatively small. Finally, since our monitoring hospitals do not include most isolated hospitals, there may be some underestimation of the incidence of preterm birth after the outbreak of COVID-19. We were only able to identify 10 women infected with the virus in the present study. A previous study from Wuhan, the area most affected by the pandemic in China, reported that from December 8, 2019 to March 20, 2020, a total of 68 pregnancies diagnosed with COVID-19 were delivered during the study period, accounting for 0.56% of all deliveries during the same time period^43^. Therefore, we believe this bias will not greatly impact the overall trend of preterm birth rate from a population perspective. In conclusion, our results show that the COVID-19 mitigation measures in China were associated with an immediate reduction in the preterm birth rate. These findings suggest that some preterm births are preventable, although its underlying the mechanism needs to be elucidated. We also call attention to the need for equitable access to health services. Further exploration of possible mechanisms is required to inform the development of novel preventive strategies aimed at mitigating the global burden of preterm birth.

## Methods

### Data and study population

We extracted all available data from the NMNMSS for the period of January 1, 2012 to December 31, 2020. The NMNMSS collects the sociodemographic and obstetric information of pregnant and postpartum women visiting obstetric departments from the time of admission to discharge. The collected data included the patients’ names, hospital code, age, education level, marital status, number of antenatal visits, date of delivery, gestational age at delivery, birth status, mode of delivery, number of foetuses, and maternal complications (at any time during hospitalisation). The detailed sampling method used has been described elsewhere^44,45^. The NMNMSS covers 438 member hospitals each assisting more than 1000 deliveries annually. The member hospitals are situated across 326 urban districts and rural counties in 30 provinces in mainland China. We restricted our study population to pregnancies greater than or equal to 28 weeks of gestation or a birthweight of 1000 g or more birthweight according to the definition of the perinatal period in China^28^.

All member facilities are required to directly report maternal questionnaires via the online NMNMSS platform. The doctors responsible for pregnancy care are responsible for completing the maternal questionnaire according to the patient’s medical records. An associate director of the obstetric department verifies and reviews the data at the primary level, investigates missing reports, aggregates the data, and reports to the NMNMSS. The county-, prefecture-, and provincial-level maternal and child healthcare hospitals oversee and verify the quality of data using random samples at the secondary, tertiary, and quaternary level, respectively. Finally, the National Office for Maternal and Child Health Surveillance conducts a data quality check at the quinary level. For institutions that fail to meet the data quality requirements, we asked them to record supplementary data. All data provided to us were de-identified. This study was approved by the ethics committee of West China Second University Hospital (Protocol ID:2012008).

### Timeframe of exposure to COVID-19 mitigation measures

To control the COVID-19 outbreak, China banned travel to and from Wuhan City on January 23, 2020; all provinces, municipalities, and autonomous regions in mainland China subsequently activated a Level 1 response over the following week^24,25^. Subsequently, China entered the implementation stage of strict epidemic mitigation measures after February 2020. Therefore, we used February 2020 as an important cut-off point in the present study. We divided the study period into two parts: a baseline stage (January 1, 2012 to January 31, 2020) and the intervention stage (February 1, 2020 to December 31, 2020).

### Outcome and data definition

The primary outcome was the monthly preterm birth rate, defined as the number of preterm births per 100 live births per month. In accordance with the criteria recommended by the World Health Organization^46^, we defined preterm birth as a delivery prior to 37 completed weeks of gestation, and further categorised it into very preterm (28–31 weeks), moderate preterm (32–33 weeks), and late preterm (34–36 weeks) birth. Preterm birth rates were calculated for each month and for each of the two stages (baseline and the intervention stage). In addition, we categorised preterm birth into (1) spontaneous labour with intact membranes, (2) preterm premature rupture of the membranes, and (3) labour induction or caesarean delivery based on maternal or foetal indications^2^; subsequently, we combined (1) and (2) as spontaneous preterm birth, and treated (3) as iatrogenic preterm birth. The secondary outcome was the monthly stillbirth rate, defined as the number of stillbirths (≥28 weeks of gestation) per 100 births per month. We defined term births as a delivery that occurred between 37 and 42 completed weeks of gestation, while births that occurred after 42 completed weeks of gestation were defined as post-term births. Gestational age is estimated based on the last menstrual period or ultrasound examination when the date of the last menstrual period is unknown^47^. We selected confounding factors based on the factors shown to be associated with preterm births in the scientific literature, including sociodemographic characteristics (maternal region, age, education level, parity, and type of pregnancy) and obstetric factors (presence of eclampsia)^2^. We defined the types of pregnancy as singleton and multiple births. As detailed elsewhere, the customary definitions of maternal region (rural and city) and maternal education level (illiteracy, primary school, middle school, college, or higher) were used^44^. The definition of city and rural areas is based on the hospital’s location.

Moreover, women who were illiterate, or had only primary school education, who were unmarried, or who had fewer than five antenatal visits were defined as the disadvantaged group. On the contrary, women with middle school or above education, married, and had more than five antenatal visits were defined as the advantaged group.

### Statistical analyses

#### Assessing the representativeness of NMNMSS data

We displayed the number of monthly births in the NMNMSS databases from January 1, 2012 to December 31, 2020 and calculated the ratio of annual monitored births to the national births and the ratio of monthly monitored births to the annual monitored births.

#### Estimating the linear trend of preterm birth rate and stillbirth rate

We examined whether there was a linear underlying time trend of preterm birth-/-stillbirth rates using the Cochran Armitage test by the PROC FREQ procedure in SAS.

#### Exploring any attribution of COVID-19 mitigation measures on preterm birth rate and stillbirth rate

We aggregated NMNMSS data down to a monthly level, then we tested whether the COVID-19 mitigation measures would specifically impact preterm birth rates and stillbirth rates. Singleton and multiple pregnancies were analysed separately, considering the inherent increased risk of preterm birth in multiple pregnancies. Interrupted time series analysis (ITSA) was used to examine changes in these rates per month over the baseline period (January 1, 2012 to January 31, 2020) and the intervention stage (February 1, 2020 to December 31, 2020). Potential confounders included in the preterm birth rate model were maternal age, parity, education level, and the presence of eclampsia, while maternal age, parity, and the presence of scar uterus were included in stillbirth rate model. Further, after comparing the regression results with and without the adjustments for seasonality, we observed the confidence intervals overlap, and the slope values are very similar. Taking the rate of preterm birth in singletons for example, an immediate reduction of −0.64% (95% CI:−1.04% to −0.24%, *p* = 0.002) was subsequently observed in the first month when implementing the COVID-19 mitigation measures when seasonal effects are not considered, while an immediate reduction of −0.68% (95% CI:−1.09% to −0.26%, *p* = 0.002) was observed when seasonality is included as a fixed effect. Finally, a categorical variable for month was included as fixed-effect to each ITSA model to adjust for any seasonality in the data, which based on that previous studies have also confirmed the scientific soundness of this correction method^48^. We specified the following model to estimate the trend in preterm birth and stillbirth rates following the implementation stage of COVID-‍19 mitigation measures:

1 $${Y}_{t}={\upbeta }_{0}+{\upbeta }_{1} \times {{{{{{\rm{time}}}}}}\, {{{{{\rm{before}}}}}}\; {{{{{\rm{intervention}}}}}}}+{\upbeta }_{2} \times {{{{{\rm{intervention}}}}}}+{\upbeta }_{3} \\ \times {{{{{{\rm{time}}}}}}\; {{{{{\rm{after}}}}}}\; {{{{{\rm{intervention}}}}}}}+{\upbeta }_{4} \times {{{{{\rm{month}}}}}}+{\upbeta }_{5} \times {{{{{\rm{covariates}}}}}}+{\upvarepsilon }_{t}$$

Here, *Y*_t_ is the preterm birth rate-/-stillbirth rate per month; *t* is a continuous variable indicating time in months, and *intervention* is an indicator for *t* occurring before (intervention = 0) or after (intervention = 1) the cap. In this model, *β*_0_ estimates the baseline level of the outcome; *β*_1_ is interpreted as the change in outcome associated with a time unit increase; *β*_2_ estimates the level of change in the preterm birth rate/stillbirth rate immediately after the intervention; and *β*_3_ estimates the slope change of the mean monthly preterm birth rate/stillbirth rate after the intervention, compared with the trend before the intervention^49^. Considering the temporary impact of COVID-19 mitigation measures, we considered significant *p*-values in *β*_*2*_ to indicate an immediate intervention effect rather than in *β*_*3*_ which indicates an intervention effect over time^50–53^. We first fitted an ordinary least squares model with a specified lag (0) and subsequently tested for autocorrelation in the error distribution. Thereafter, we identified the corresponding lag according to the autocorrelation. For a more in-depth understanding as to whether COVID-19 mitigation measures have aggravated existing health inequalities within populations, we further estimated the change in the preterm birth rate over time as per SES. Furthermore, in order to observe the difference in preterm birth-/ -stillbirth rates between a theoretical assumption that mitigation measures had not occurred and actual lockdown circumstances, we developed counterfactual scenarios. A predicted rate of preterm birth/stillbirth were calculated using the Betas from primary model (this produces Newey-West standard errors for coefficients estimated by ordinary least-squares regression), but setting the lockdown dummy at ‘0’ for the entire study period. This counterfactual preterm birth/stillbirth rate represented the theoretical rate of preterm birth, had COVID-19 mitigation measures not been implemented.

A range of sensitivity analyses were conducted to evaluate the robustness of the results from our primary analysis. First, we confirmed the changes in the preterm birth rates at 1-, 2-, 3-, and 6-month lags after the implementation of COVID-19 mitigation measures. Second, we restricted the analysis to pregnancies in Wuhan to confirm the changes in the preterm birth and stillbirth rates over the COVID-19 mitigation period. Third, we performed a sensitivity analysis using 2016–‍2020 data instead of 2012–2020 data, allowing us to assess whether the results differed when the balance between the time points changed. Finally, given the timing of the outbreak, we redefined December 2019 and January 2020 as the cut-off points of the interrupted time series analysis to explore the change in the preterm birth rate. The measures at this stage were not widespread or stringent. Analyses were performed using Stata Statistical Software (Release 16; StataCorp, College Station, TX, USA), and SAS statistical software version 9.4 (SAS Institute Inc., NC, USA). All *p*-values were two-sided, and statistical significance was set at *p* < 0.05.

### Reporting summary

Further information on research design is available in the Nature Research Reporting Summary linked to this article.

## Supplementary information

Supplementary Information

Description of Additional Supplementary Files

Supplementary Software 1

Reporting Summary

## Data Availability

The datasets used in the study comprises individual-level sensitive information from national register data. According to the Chinese data protection legislation, the authors are not allowed to share these sensitive data directly upon request. However, the data are available for research upon a necessary request to the National Maternal and Child Health Surveillance Office of China (email:zhujun028@163.com). The request should meet the framework of the Chinese data protection legislation and any required permission from the National Health Commission of the People’s Republic of China. The data request must specify the research purpose, specific method, expected results, results sharing plan, and whether it involves ethics and other details. Expect a time frame of at least 6–8 months for data requests to be processed.

## Code availability

The codes used for the statistical models in the main text are recorded as Supplementary Software 1 (including a Stata code file of ITSA analysis among singleton births (multiple births are similar), a SAS code files for Cochran Armitage test, and an introduction file of variables in original datasets) All codes are available from the corresponding author upon request.

## References

[1] WHO: recommended definitions, terminology, and format for statistical tables related to the perinatal period and use of a new certificate for cause of perinatal deaths. Modifications recommended by FIGO as amended October 14, 1976. *Acta Obstet. Gynecol. Scand*. **56**, 247–253 (1977). 560099

[2] Goldenberg RL, Culhane JF, Iams JD, Romero R (2008). Epidemiology and causes of preterm birth. Lancet.

[3] Chawanpaiboon S (2019). Global, regional, and national estimates of levels of preterm birth in 2014: a systematic review and modelling analysis. Lancet Glob. Health.

[4] Deng K (2021). Preterm births in China between 2012 and 2018: an observational study of more than 9 million women. Lancet Glob. Health.

[5] Singer LT (1999). Maternal psychological distress and parenting stress after the birth of a very low-birth-weight infant. JAMA.

[6] McCormick MC (1985). The contribution of low birth weight to infant mortality and childhood morbidity. N. Engl. J. Med.

[7] Bérard A, Le Tiec M, De Vera MA (2012). Study of the costs and morbidities of late-preterm birth. Arch. Dis. Child Fetal Neonatal Ed..

[8] Iams JD, Romero R, Culhane JF, Goldenberg RL (2008). Primary, secondary, and tertiary interventions to reduce the morbidity and mortality of preterm birth. Lancet.

[9] Green J, Petty J, Whiting L, Fowler C (2021). Exploring modifiable risk-factors for premature birth in the context of COVID-19 mitigation measures: A discussion paper. J. Neonatal Nurs..

[10] Chmielewska B (2021). Effects of the COVID-19 pandemic on maternal and perinatal outcomes: a systematic review and meta-analysis. Lancet Glob. Health.

[11] Hedermann G (2021). Danish premature birth rates during the COVID-19 lockdown. Arch. Dis. Child Fetal Neonatal Ed..

[12] Richter, F. et al. Neonatal outcomes during the COVID-19 pandemic in New York City. *Pediatr. Res.*, 1–3, 10.1038/s41390-021-01513-7 (2021). 10.1038/s41390-021-01513-7 PMC8025442 33828229

[13] Philip, R. K. et al. Unprecedented reduction in births of very low birthweight (VLBW) and extremely low birthweight (ELBW) infants during the COVID-19 lockdown in Ireland: a ‘natural experiment’ allowing analysis of data from the prior two decades. *BMJ Glob. Health* **5**, 10.1136/bmjgh-2020-003075 (2020). 10.1136/bmjgh-2020-003075 PMC7528371 32999054

[14] Matheson A (2021). Prematurity rates during the Coronavirus disease 2019 (COVID-19) Pandemic Lockdown in Melbourne, Australia. Obstet. Gynecol..

[15] Main EK (2021). Singleton preterm birth rates for racial and ethnic groups during the coronavirus disease 2019 pandemic in California. Am. J. Obstet. Gynecol..

[16] Huseynova R (2021). Prevalence of preterm birth rate during COVID-19 lockdown in a tertiary care hospital, Riyadh. Cureus.

[17] Handley SC (2021). Changes in preterm birth phenotypes and stillbirth at 2 Philadelphia hospitals during the SARS-CoV-2 pandemic, March–June 2020. JAMA.

[18] Caniglia EC (2021). Modest reduction in adverse birth outcomes following the COVID-19 lockdown. Am. J. Obstet. Gynecol..

[19] Been JV (2020). Impact of COVID-19 mitigation measures on the incidence of preterm birth: a national quasi-experimental study. Lancet Public Health.

[20] Maeda Y (2021). Trends in intensive neonatal care during the COVID-19 outbreak in Japan. Arch. Dis. Child Fetal Neonatal Ed..

[21] Bian, Z., Qu, X., Ying, H. & Liu, X. Are COVID-19 mitigation measures reducing preterm birth rate in China? *BMJ Glob. Health* **6**, 10.1136/bmjgh-2021-006359 (2021). 10.1136/bmjgh-2021-006359 PMC8361681 34385161

[22] Lin TT (2021). COVID-19 lockdown increased the risk of preterm birth. Front. Med..

[23] Li Z (2020). Active case finding with case management: the key to tackling the COVID-19 pandemic. Lancet.

[24] *National Public Health Emergency Response Protocol 2006*, <http://www.gov.cn/yjgl/2006-02/26/content_211654.htm>.

[25] Tian H (2020). An investigation of transmission control measures during the first 50 days of the COVID-19 epidemic in China. Science.

[26] China, N. H. C. o. t. P. s. R. o. *Novel coronavirus pneumonia infection prevention and control program (second edition)*, <http://www.nhc.gov.cn/xcs/zhengcwj/list_gzbd_9.shtml> (2020).

[27] China, N. H. C. o. t. P. s. R. o. *Notice on strict prevention of novel coronavirus infection by means of transportation*, <http://www.nhc.gov.cn/xcs/zhengcwj/202001/e5e8c983baba4c1589512e6c99fdaa4e.shtml> (2020).

[28] Xing Xie, W. G. *Obstetrics and gynecology*. 142 (People’s Health Publishing 2013).

[29] Waldenström U, Cnattingius S, Vixner L, Norman M (2017). Advanced maternal age increases the risk of very preterm birth, irrespective of parity: a population-based register study. BJOG.

[30] Huang QS (2021). Impact of the COVID-19 nonpharmaceutical interventions on influenza and other respiratory viral infections in New Zealand. Nat. Commun..

[31] Burns J (2021). COVID-19 mitigation measures and nitrogen dioxide—A quasi-experimental study of air quality in Munich, Germany. Atmos. Environ..

[32] Zhang X, Kramer MS (2012). The rise in singleton preterm births in the USA: the impact of labour induction. BJOG.

[33] MacDorman MF, Declercq E, Zhang J (2010). Obstetrical intervention and the singleton preterm birth rate in the United States from 1991–2006. Am. J. Public Health.

[34] Richards JL (2016). Temporal trends in late preterm and early term birth rates in 6 high-income countries in North America and Europe and association with clinician-initiated obstetric interventions. JAMA.

[35] Chen C (2019). Preterm birth in China between 2015 and 2016. Am. J. Public Health.

[36] Ma L (2020). Knowledge, beliefs/attitudes, and practices of rural residents in the prevention and control of COVID-19: An Online Questionnaire Survey. Am. J. Trop. Med Hyg..

[37] Wiersinga WJ, Rhodes A, Cheng AC, Peacock SJ, Prescott HC (2020). Pathophysiology, transmission, diagnosis, and treatment of coronavirus disease 2019 (COVID-19): a review. JAMA.

[38] Abrams EM, Szefler SJ (2020). COVID-19 and the impact of social determinants of health. Lancet Respir. Med..

[39] Anderson G, Frank JW, Naylor CD, Wodchis W, Feng P (2020). Using socioeconomics to counter health disparities arising from the covid-19 pandemic. BMJ.

[40] Macaulay S, Buchmann EJ, Dunger DB, Norris SA (2019). Reliability and validity of last menstrual period for gestational age estimation in a low-to-middle-income setting. J. Obstet. Gynaecol. Res.

[41] Hoffman CS (2008). Comparison of gestational age at birth based on last menstrual period and ultrasound during the first trimester. Paediatr. Perinat. Epidemiol..

[42] Savitz DA (2002). Comparison of pregnancy dating by last menstrual period, ultrasound scanning, and their combination. Am. J. Obstet. Gynecol..

[43] Chen L (2020). Clinical characteristics of pregnant women with Covid-19 in Wuhan, China. N. Engl. J. Med..

[44] Liang J (2018). Relaxation of the one child policy and trends in caesarean section rates and birth outcomes in China between 2012 and 2016: observational study of nearly seven million health facility births. BMJ.

[45] Xiong T (2020). Association between ambient temperature and hypertensive disorders in pregnancy in China. Nat. Commun..

[46] Swaminathan A, Fell DB, Regan A, Walker M, Corsi DJ (2020). Association between interpregnancy interval and subsequent stillbirth in 58 low-income and middle-income countries: a retrospective analysis using Demographic and Health Surveys. Lancet Glob. Health.

[47] Zhu J (2016). Sociodemographic and obstetric characteristics of stillbirths in China: a census of nearly 4 million health facility births between 2012 and 2014. Lancet Glob. Health.

[48] Bhaskaran K, Gasparrini A, Hajat S, Smeeth L, Armstrong B (2013). Time series regression studies in environmental epidemiology. Int J. Epidemiol..

[49] Bernal JL, Cummins S, Gasparrini A (2017). Interrupted time series regression for the evaluation of public health interventions: a tutorial. Int J. Epidemiol..

[50] Linden A, Adams JL (2011). Applying a propensity score-based weighting model to interrupted time series data: improving causal inference in programme evaluation. J. Eval. Clin. Pr..

[51] A L (2015). Conducting interrupted time-series analysis for single- and multiple-group comparisons. Stata J..

[52] Mu Y (2021). The trends and associated adverse maternal and perinatal outcomes of labour neuraxial analgesia among vaginal deliveries in China between 2012 and 2019: a real-world observational evidence. BMC Med..

[53] Li HT (2019). Association of China’s universal two-child policy with changes in births and birth-related health factors: national, descriptive comparative study. BMJ.

