# Supplementary material for: Interrupted-time-series analysis of the immediate impact of COVID-19 mitigation measures on preterm birth in China
Source: Nat Commun. 2022 Sep 3;13:5190. doi: 10.1038/s41467-022-32814-y (PMC9440464; doi:10.1038/s41467-022-32814-y)
Supplement: Supplementary file 1 — Supplementary Information [file 41467_2022_32814_MOESM1_ESM.pdf]

Supplementary Information

Title: Interrupted-time-series analysis of the immediate impact of COVID-19 mitigation measures on preterm birth in China

Authors: Yanxia Xie et al.

Included Files

Supplementary Fig. 1-6

Supplementary Table 1-3

Supplementary Figures

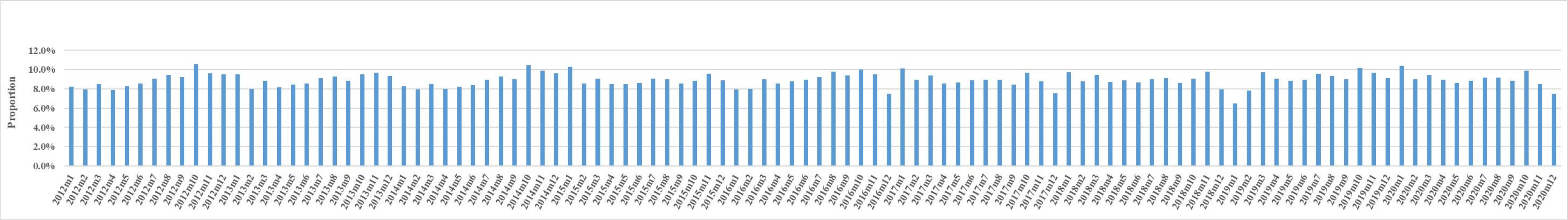

Supplementary Fig. 1 The proportion of monthly monitored births to the annual monitored births in NMNMSS, 2012-2020.

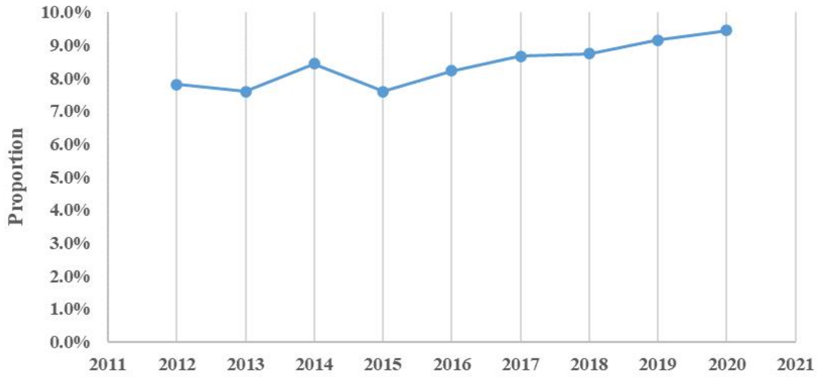

Supplementary Fig. 2 The proportion of annual monitored births in NMNMSS to the national births in China, 2012-2020.

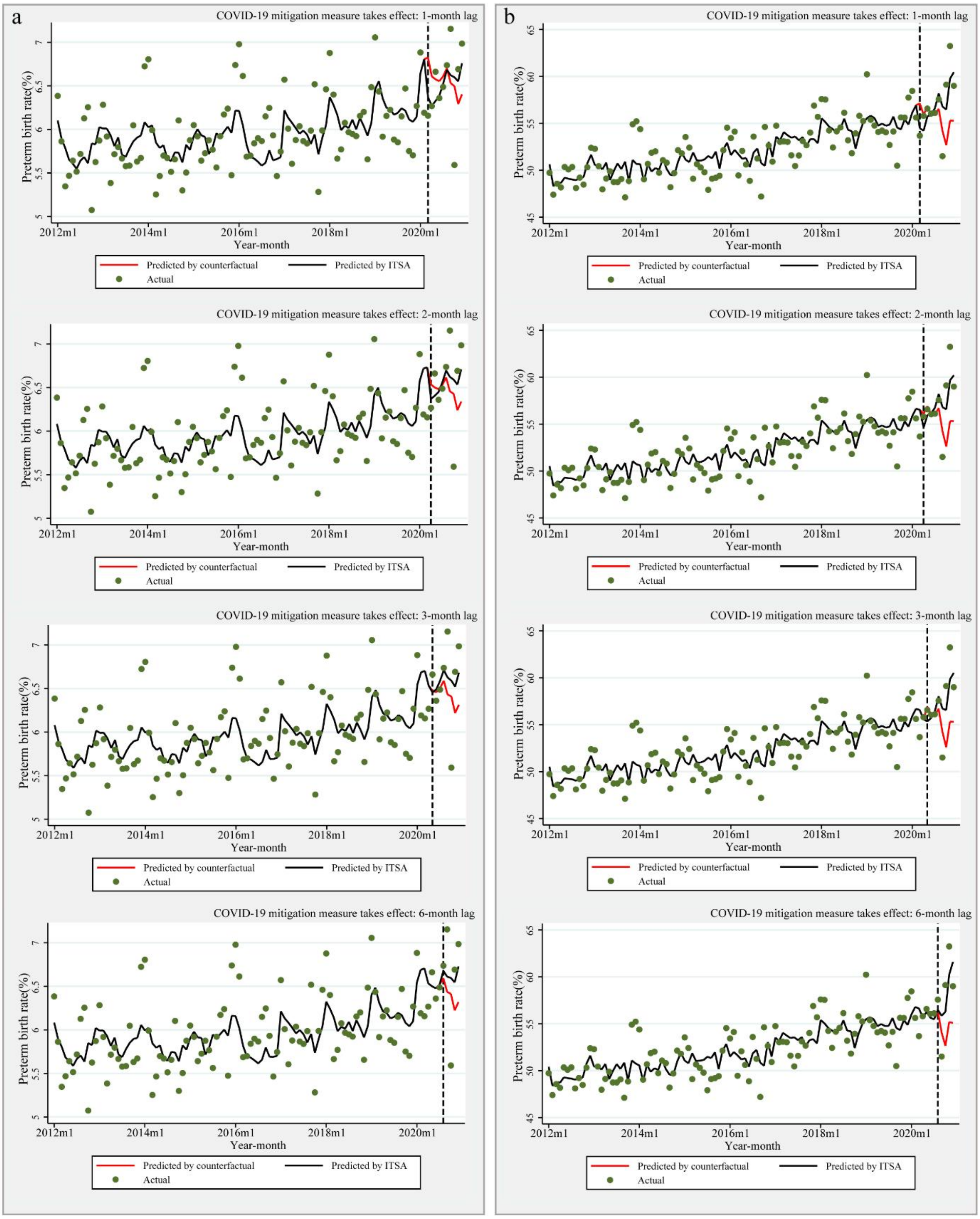

Supplementary Fig. 3 Interrupted time series analysis (ITSA) of preterm birth rates at 1-, 2-, 3-, and 6-month lags after the implementation of COVID-19 mitigation measures among singleton and multiple births in China, 2012-2020. (a) singleton births; (b) multiple births. Dots indicate true monthly stillbirth rates; solid lines indicate the mean of estimated stillbirth rates per month by ITSA model, and red solid lines indicate the mean of estimated preterm birth rates per month assuming that COVID-19 mitigation measures had not occurred by ordinary least-squares regression with Newey-West standard errors. Baseline: from January 1, 2012 to January 31, 2020; Intervention stage:

from February 1, 2020 to December 31, 2020. Intercept change: change in level compared with the previous stage; slope change: change in trend compared with the previous stage, per month. COVID-19: coronavirus disease 2019. The *p* values are two-sided.

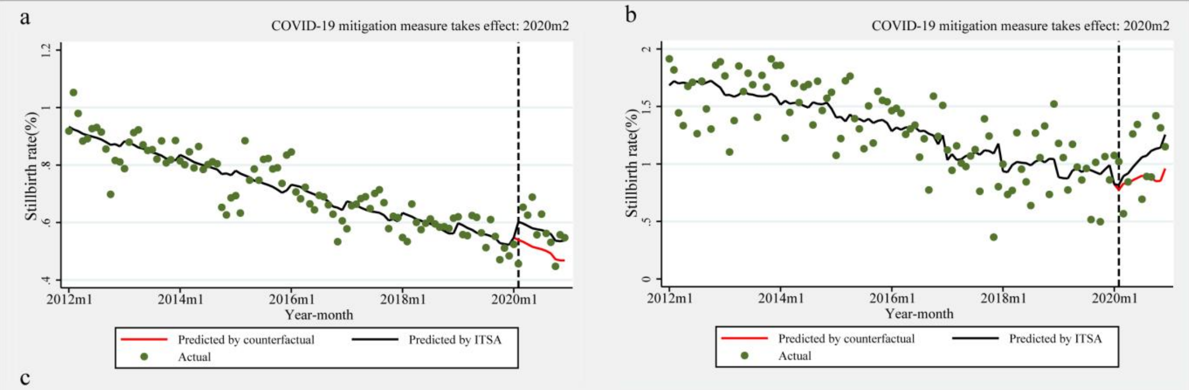

c

	Singleton births			Multiple births		
	β (Coefficient)	*P*-value	95%CI	β (Coefficient)	*P*-value	95%CI
Baseline intercept	1.05	<0.001	0.77~1.34	2.07	<0.001	1.38~2.77
Baseline slope	-0.004	<0.001	-0.005~-0.003	-0.007	<0.001	-0.011~-0.004
Intervention-Intercept change	0.060	0.253	-0.044~0.164	0.052	0.742	-0.262~0.366
Intervention-Slope change	0.001	0.924	-0.015~0.017	0.027	0.260	-0.020~0.074

Supplementary Fig. 4 Interrupted time series analysis (ITSA) of stillbirth rates among singletons and multiples in China, 2012-2020. (a) singleton births; (b) multiple births; (c) the result of ITSA across different type pregnancies. Dots indicate true monthly stillbirth rates; solid lines indicate the mean of estimated stillbirth rates per month by ITSA model, and red solid lines indicate the mean of estimated preterm birth rates per month assuming that COVID-19 mitigation measure had not occurred by ordinary least-squares regression with Newey-West standard errors. Baseline: from January 1, 2012 to January 31, 2020; Intervention stage: from February 1, 2020 to December 31, 2020. Intercept change: change in level compared with the previous stage; slope change: change in trend compared with the previous stage, per month. CI: confidence interval; COVID-19: coronavirus disease 2019. The *p* values are two-sided.

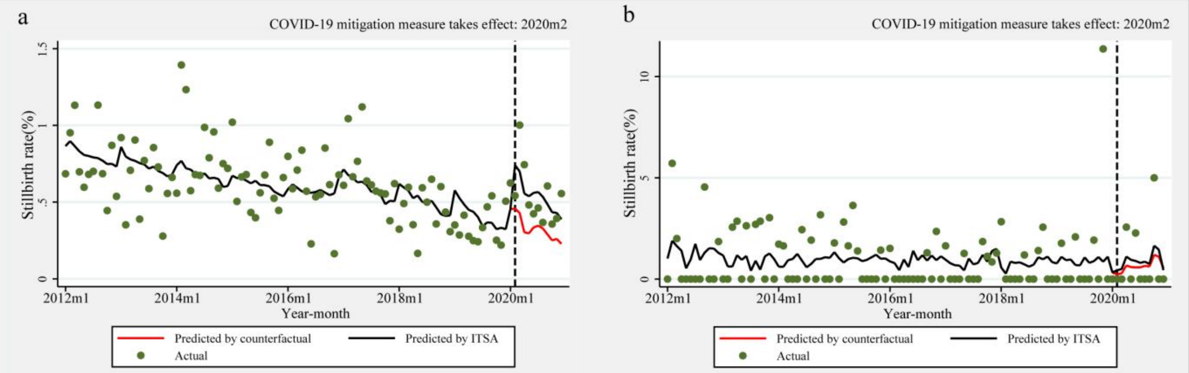

c

	Singleton births			Multiple births		
	**β (Coefficient)**	***P*-value**	**95%CI**	**β (Coefficient)**	***P*-value**	**95%CI**
Baseline intercept	0.79	<0.001	0.61~0.96	1.07	0.039	0.06~2.08
Baseline slope	-0.006	<0.001	-0.009~-0.003	-0.007	0.536	-0.028~0.014
Intervention-Intercept change	0.279	0.050	0.000~0.557	0.519	0.438	-0.805~1.844
Intervention-Slope change	-0.013	0.546	-0.056~0.030	-0.041	0.676	-0.234~0.154

Supplementary Fig. 5 Interrupted time series analysis (ITSA) of stillbirth rates among singleton and multiple births in Wuhan, China, 2012-2020. (a) singleton births; (b) multiple births; (c) the result of ITSA across different type pregnancies. Dots indicate true monthly stillbirth rates; solid lines indicate the mean of estimated stillbirth rates per month by ITSA model, and red solid lines indicate the mean of estimated preterm birth rates per month assuming that COVID-19 mitigation measure had not occurred by ordinary least-squares regression with Newey-West standard errors. Baseline: from January 1, 2012 to January 31, 2020; Intervention stage: from February 1, 2020 to December 31, 2020. Intercept change: change in level compared with the previous stage; slope change: change in trend compared with the previous stage, per month. CI: confidence interval; COVID-19: coronavirus disease 2019. The *p* values are two-sided.

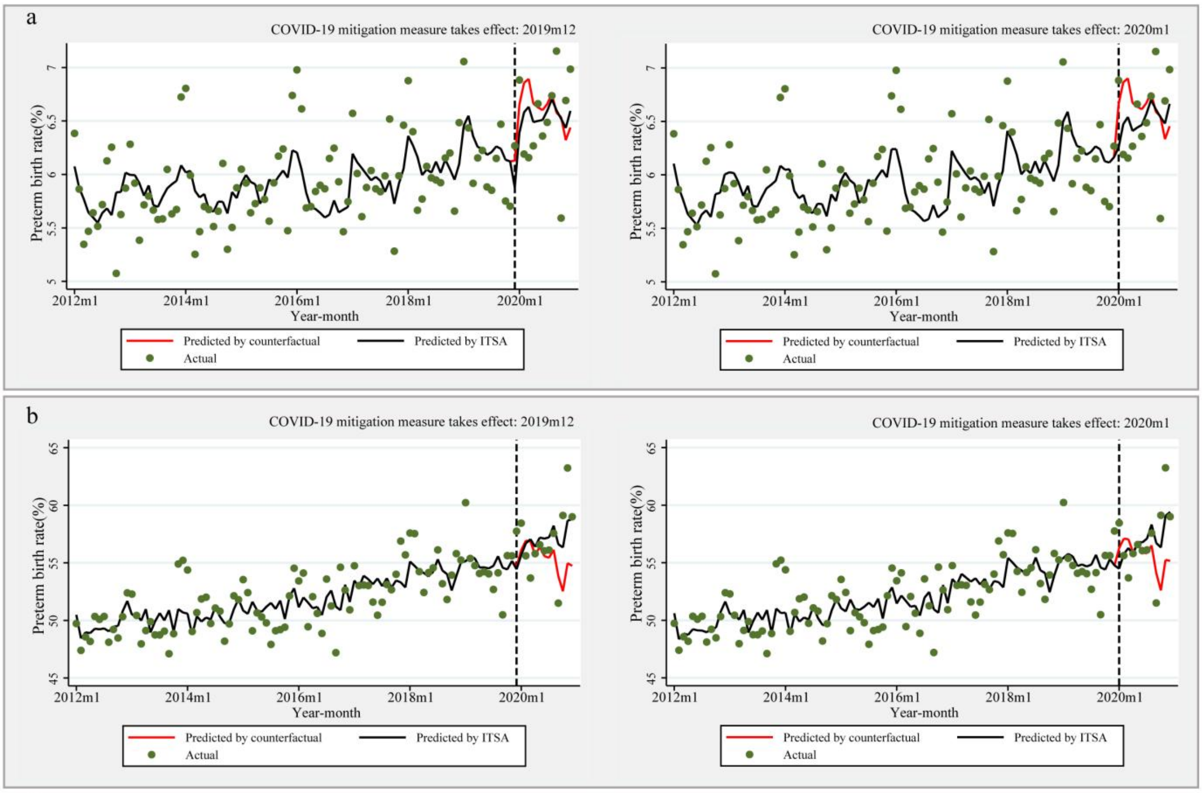

c

	Singleton births			Multiple births		
	β (Coefficient)	P-value	95%CI	β (Coefficient)	P-value	95%CI
2019.12						
Baseline intercept	8.45	<0.001	5.53~11.37	46.45	<0.001	35.42~57.48
Baseline slope	0.027	0.011	0.006~0.047	0.035	0.233	-0.023~0.092
Intervention-Intercept change	-0.299	0.340	-0.916~0.319	-0.188	0.920	-3.913~3.537
Intervention-Slope change	0.036	0.230	-0.023~0.095	0.348	0.206	-0.194~0.889
2020.1						
Baseline intercept	8.80	<0.001	5.62~11.97	48.57	<0.001	37.82~59.33
Baseline slope	0.029	0.008	0.007~0.050	0.047	0.100	-0.009~0.102
Intervention-Intercept change	-0.463	0.117	-1.043~0.118	-1.467	0.365	-4.665~1.730
Intervention-Slope change	0.059	0.053	-0.001~0.118	0.541	0.057	-0.017~1.098

Supplementary Fig. 6 Interrupted time series analysis (ITSA) of preterm birth rates when the time cut-off is assumed to be December 2019 or January 2020 in China, 2012-2020. (a) singleton births; (b) multiple births; (c) the result of ITSA across different type pregnancies. Dots indicate true monthly stillbirth rates; solid lines indicate the mean of estimated preterm birth rates per month by ITSA model, and red solid lines indicate the mean of estimated preterm birth rates per month assuming that COVID-19 mitigation measure had not occurred by ordinary least-squares regression with Newey-West standard errors. Baseline: from January 1, 2012 to January 31, 2020; Intervention stage: from February 1, 2020 to December 31, 2020. Intercept change: change in level compared with the previous stage; slope change: change in trend compared with the previous stage, per month. CI: confidence interval; COVID-19: coronavirus disease 2019. The *p* values are two-sided.

Supplementary Table 1. Interrupted time series analysis of monthly preterm birth rate across different lag months in singleton and multiple pregnancies in China, 2012-2020.

	Singleton births			Multiple births		
	**β (Coefficient)**	**P-value**	**95%CI**	**β (Coefficient)**	**P-value**	**95%CI**
**1-month lag**						
Baseline intercept	8.38	<0.001	5.75~11.01	50.22	<0.001	39.83~60.62
Baseline slope	0.026	0.005	0.008~0.044	0.054	0.048	0.001~0.108
Intervention-Intercept change	-0.45	0.009	-0.786~-0.115	-2.404	0.009	-4.202~-0.606
Intervention-Slope change	0.087	0.001	0.035~0.139	0.874	<0.001	0.426~1.323
**2-month lag**						
Baseline intercept	8.03	<0.001	5.46~10.60	48.78	<0.001	38.21~59.36
Baseline slope	0.023	0.014	0.005~0.041	0.044	0.120	-0.012~0.100
Intervention-Intercept change	-0.191	0.270	-0.531~0.150	-0.902	0.389	-2.970~1.166
Intervention-Slope change	0.067	0.022	0.010~0.125	0.792	0.004	0.266~1.317
**3-month lag**						
Baseline intercept	7.88	<0.001	5.40~10.35	49.25	<0.001	39.95~59.56
Baseline slope	0.022	0.016	0.004~0.039	0.046	0.090	-0.007~0.100
Intervention-Intercept change	-0.043	0.832	-0.442~0.356	-0.755	0.460	-2.774~1.265
Intervention-Slope change	0.056	0.147	-0.020~0.131	0.924	0.002	0.357~1.491
**6-month lag**						
Baseline intercept	7.86	<0.001	5.64~10.08	50.04	<0.001	40.68~59.39
Baseline slope	0.022	0.005	0.007~0.036	0.051	0.032	0.004~0.098
Intervention-Intercept change	0.087	0.740	-0.430~0.603	0.335	0.821	-2.593~3.264
Intervention-Slope change	0.077	0.410	-0.107~0.261	1.590	0.010	0.384~2.796

The *p* values are two-sided; CI: confidence interval; Baseline: January 1, 2012 to January 31, 2020; Intervention stage:February 1, 2020 to December 31, 2020.

Supplementary Table 2. Interrupted time series analysis of monthly preterm birth rate in singleton and multiple pregnancies in China, 2016-2020.

	**Singleton births**			**Multiple births**		
	**β (Coefficient)**	***P*-value**	**95%CI**	**β (Coefficient)**	***P*-value**	**95%CI**
**Preterm**						
Baseline intercept	17.71	<0.001	12.54~22.87	61.37	<0.001	42.12~80.62
Baseline slope	0.023	0.037	0.001~0.045	0.147	0.011	0.035~0.259
Intervention-Intercept change	-0.594	0.008	-1.029~-0.159	-4.076	<0.001	-5.725~-2.426
Intervention-Slope change	0.130	<0.001	0.089~0.171	0.774	<0.001	0.471~1.077
**Very preterm**						
Baseline intercept	1.01	0.003	0.37~1.65	2.74	0.344	-3.01~8.48
Baseline slope	-0.001	0.415	-0.004~0.002	0.015	0.481	-0.026~0.055
Intervention-Intercept change	0.006	0.802	-0.041~0.053	0.030	0.955	-1.033~1.094
Intervention-Slope change	0.012	<0.001	0.007~0.016	0.032	0.824	-0.257~0.322
**Moderate preterm**						
Baseline intercept	1.51	<0.001	0.87~2.16	8.19	0.072	-0.75~17.13
Baseline slope	-0.000	0.983	-0.003~0.004	0.049	0.087	-0.007~0.105
Intervention-Intercept change	-0.046	0.125	-0.104~0.013	-1.279	0.025	-2.391~-0.167
Intervention-Slope change	0.012	0.001	0.005~0.019	0.095	0.434	-0.146~0.336
**Late preterm**						
Baseline intercept	15.18	<0.001	10.76~19.60	50.44	<0.001	37.97~62.91
Baseline slope	0.024	0.005	0.008~0.041	0.084	0.037	0.005~0.162
Intervention-Intercept change	-0.555	0.001	-0.863~-0.247	-2.827	0.004	-4.729~-0.924
Intervention-Slope change	0.106	<0.001	0.078~0.134	0.648	<0.001	0.414~0.881
**Spontaneous preterm**						
Baseline intercept	11.68	<0.001	8.51~14.85	24.62	<0.001	16.05~33.21

Baseline slope	0.015	0.021	0.002~0.028	0.024	0.498	-0.046~0.094
Intervention-Intercept change	-0.434	<0.001	-0.668~-0.201	-1.868	<0.001	-2.683~-1.053
Intervention-Slope change	0.066	<0.001	0.034~0.097	0.293	<0.001	0.135~0.451
**Iatrogenic preterm**						
Baseline intercept	6.03	<0.001	3.55~8.50	36.74	<0.001	23.28~50.20
Baseline slope	0.008	0.087	-0.001~0.017	0.123	0.004	0.042~0.204
Intervention-Intercept change	-0.160	0.111	-0.358~0.038	-2.208	0.060	-4.514~0.098
Intervention-Slope change	0.064	<0.001	0.042~0.086	0.481	0.032	0.042~0.921

The p values are two-sided; CI: confidence interval; Baseline: January 1, 2012 to January 31, 2020; Intervention stage:February 1, 2020 to December 31, 2020.

Supplementary Table 3. Interrupted time series analysis of monthly preterm birth rate in singleton and multiple pregnancies in China, 2012-2020 (the perinatal birth definition of gestational age above 22w was used).

	**Singleton births**			**Multiple births**		
	**β (Coefficient)**	***P*-value**	**95% CI**	**β (Coefficient)**	***P*-value**	**95% CI**
**Preterm**						
Baseline intercept	9.13	<0.001	6.25~12.01	51.34	<0.001	40.73~61.96
Baseline slope	0.032	0.001	0.013~0.051	0.066	0.019	0.011~0.120
Intervention-Intercept change	-0.709	0.001	-1.128~-0.289	-3.053	<0.001	-4.810~-1.295
Intervention-Slope change	0.104	<0.001	0.056~0.152	0.827	<0.001	0.406~1.248
**Extremely preterm**						
Baseline intercept	0.18	<0.001	0.09~0.27	0.45	0.504	-0.88~1.76
Baseline slope	0.002	<0.001	0.001~0.002	0.012	<0.001	0.005~0.018
Intervention-Intercept change	-0.03	0.021	-0.055~-0.005	-0.505	0.006	-0.862~-0.147
Intervention-Slope change	0.009	<0.001	0.005~0.012	0.096	0.006	0.028~0.165
**Very preterm**						
Baseline intercept	0.78	<0.001	0.47~1.09	4.66	0.006	1.38~7.93
Baseline slope	0.003	0.027	0.000~0.005	0.010	0.293	-0.009~0.030
Intervention-Intercept change	-0.025	0.341	-0.077~0.027	0.175	0.731	-0.829~1.178
Intervention-Slope change	0.005	0.203	-0.003~0.013	0.023	0.859	-0.236~0.282
**Moderate preterm**						
Baseline intercept	1.06	<0.001	0.66~1.47	6.22	0.019	1.06~11.39
Baseline slope	0.003	0.069	0.000~0.005	-0.002	0.899	-0.026~0.023
Intervention-Intercept change	-0.073	0.005	-0.124~-0.023	-0.394	0.335	-1.200~0.413
Intervention-Slope change	0.008	0.003	0.003~0.013	0.105	0.110	-0.024~0.234
**Late preterm**						
Baseline intercept	7.11	<0.001	4.55~9.67	40.01	<0.001	31.30~48.73
Baseline slope	0.025	0.002	0.009~0.041	0.045	0.061	-0.002~0.093

Intervention-Intercept change	-0.581	0.001	-0.925~-0.236	-2.329	<0.001	-3.558~1.100
Intervention-Slope change	0.082	<0.001	0.051~0.114	0.602	<0.001	0.436~0.769
**Spontaneous preterm**						
Baseline intercept	5.58	<0.001	3.63~7.53	20.33	<0.001	13.67~26.98
Baseline slope	0.021	0.004	0.007~0.035	0.045	0.029	0.005~0.085
Intervention-Intercept change	-0.489	0.003	-0.805~-0.173	-1.993	0.006	-3.389~-0.597
Intervention-Slope change	0.039	0.088	-0.006~0.083	0.193	0.164	-0.080~0.465
**Iatrogenic preterm**						
Baseline intercept	3.55	<0.001	2.55~4.55	31.02	<0.001	22.40~39.63
Baseline slope	0.011	<0.001	0.005~0.017	0.021	0.392	-0.027~0.068
Intervention-Intercept change	-0.219	0.008	-0340~-0.059	-1.060	0.205	-2.708~0.590
Intervention-Slope change	0.065	<0.001	0.042~0.089	0.634	<0.001	0.287~0.982

The p values are two-sided; CI: confidence interval; Baseline: January 1, 2012 to January 31, 2020; Intervention stage:February 1, 2020 to December 31, 2020.
