# Supplementary material for: Interrupted-time-series analysis of the immediate impact of COVID-19 mitigation measures on preterm birth in China
Source: Nat Commun. 2022 Sep 3;13:5190. doi: 10.1038/s41467-022-32814-y (PMC9440464; doi:10.1038/s41467-022-32814-y)
Supplement: Supplementary file 2 — Description of Additional Supplementary Files [file 41467_2022_32814_MOESM2_ESM.pdf]

Description of Additional Supplementary Files

File Name: Supplementary Software 1

Description: Includes a Stata code file of ITSA analysis among singleton births (multiple births are similar), a SAS code file for Cochran Armitage test, and an introduction file of variables in original datasets.
