# Supplementary material for: Interrupted-time-series analysis of the immediate impact of COVID-19 mitigation measures on preterm birth in China
Source: Nat Commun. 2022 Sep 3;13:5190. doi: 10.1038/s41467-022-32814-y (PMC9440464; doi:10.1038/s41467-022-32814-y)
Supplement: Supplementary file 3 — Supplementary Software 1 [file 41467_2022_32814_MOESM3_ESM.zip › Code/appendix. variable explanation for code.docx]

The do file in the supplement files includes the code source for an interrupted-time-series analysis among singleton births (multiple births are similar). The “itsa” and “actest” package of Stata is needed (typing ‘ssc install itsa’ and ‘ssc install actest’ in the command window of stata). The do file runs well in the Stata 16.0 with Windows 7.

The sas file in the supplement files includes the source codes for Cochran Armitage test. The sas file runs well in the SAS 9.4 with Windows 7.

Variables used in models:

| age_35 | mother’s age, a category variable. 1>=35y, 0<35y. |
| --- | --- |
| parity | a category variable. 1multiparous, 0 nulliparous. |
| eclampsia | a category variable. 1 Yes, 0 No. |
| edu_2 | a category variable 1 High school and above, 0 middle high and below. |
| scar | a category variable. 1 Yes, 0 No. |
| month3 | fixed-effect monthly indicator variables. |
| shdw | indicators of socioeconomic status, a category variable. 1 disadvantaged, 0 advantaged. |
| monthly | A time variable in months. |
| study1 | singleton live births, a category variable. 1 Yes, 0 No. |
| study2 | perinatal birth, a category variable. 1 Yes, 0 No. |
| study3 | multiple live births, a category variable. 1 Yes, 0 No. |
| wuhan | a category variable. 1 Yes, 0 No. |
| b_21 | Number of fetus, numerical variable. |
| preterm | a category variable. 1 Yes, 0 No. |
| spontaneous_preterm | a category variable. 1 Yes, 0 No. |
| iatrogenic_preterm | a category variable. 1 Yes, 0 No. |
| very_preterm | a category variable. 1 Yes, 0 No. |
| moderate_preterm | a category variable. 1 Yes, 0 No. |
| late_preterm | a category variable. 1 Yes, 0 No. |
| stillbirth | a category variable. 1 Yes, 0 No. |
