# Supplementary material for: Interrupted-time-series analysis of the immediate impact of COVID-19 mitigation measures on preterm birth in China
Source: Nat Commun. 2022 Sep 3;13:5190. doi: 10.1038/s41467-022-32814-y (PMC9440464; doi:10.1038/s41467-022-32814-y)
Supplement: Supplementary file 4 — Reporting Summary [file 41467_2022_32814_MOESM4_ESM.pdf]

nature portfolio

Corresponding author(s): Juan Liang and Jun Zhu
Last updated by author(s): Aug 2, 2022

Reporting Summary

Nature Portfolio wishes to improve the reproducibility of the work that we publish. This form provides structure for consistency and transparency in reporting. For further information on Nature Portfolio policies, see our Editorial Policies and the Editorial Policy Checklist.

Statistics

For all statistical analyses, confirm that the following items are present in the figure legend, table legend, main text, or Methods section.

n/a	Confirmed	
☒	☐	The exact sample size (n) for each experimental group/condition, given as a discrete number and unit of measurement
☒	☐	A statement on whether measurements were taken from distinct samples or whether the same sample was measured repeatedly
☐	☒	The statistical test(s) used AND whether they are one- or two-sided<br>*Only common tests should be described solely by name; describe more complex techniques in the Methods section.*
☐	☒	A description of all covariates tested
☒	☐	A description of any assumptions or corrections, such as tests of normality and adjustment for multiple comparisons
☐	☒	A full description of the statistical parameters including central tendency (e.g. means) or other basic estimates (e.g. regression coefficient) AND variation (e.g. standard deviation) or associated estimates of uncertainty (e.g. confidence intervals)
☒	☐	For null hypothesis testing, the test statistic (e.g. F, t, r) with confidence intervals, effect sizes, degrees of freedom and P value noted<br>*Give P values as exact values whenever suitable.*
☒	☐	For Bayesian analysis, information on the choice of priors and Markov chain Monte Carlo settings
☐	☒	For hierarchical and complex designs, identification of the appropriate level for tests and full reporting of outcomes
☒	☐	Estimates of effect sizes (e.g. Cohen's d, Pearson's r), indicating how they were calculated

Our web collection on statistics for biologists contains articles on many of the points above.

Software and code

Policy information about availability of computer code

Data collection	Analyses were performed using Stata Statistical Software (Release 16; StataCorp, College Station, TX, USA).
Data analysis	Analyses were performed using Stata Statistical Software (Release 16; StataCorp, College Station, TX, USA), and SAS statistical software version 9.4 (SAS Institute Inc., NC, USA).

For manuscripts utilizing custom algorithms or software that are central to the research but not yet described in published literature, software must be made available to editors and reviewers. We strongly encourage code deposition in a community repository (e.g. GitHub). See the Nature Portfolio guidelines for submitting code & software for further information.

Data

Policy information about availability of data

All manuscripts must include a data availability statement. This statement should provide the following information, where applicable:

- Accession codes, unique identifiers, or web links for publicly available datasets
- A description of any restrictions on data availability
- For clinical datasets or third party data, please ensure that the statement adheres to our policy

Data supporting the findings of this study are available from the National Maternal and Child Health Surveillance Office of China, but the availability of these data is limited and they were used under the license of the current study and are therefore not publicly available. However, the data are available for research upon a necessary request to the National Maternal and Child Health Surveillance Office of China (email:zhujun028@163.com). The request should meet the framework of the Chinese data protection legislation and any required permission from the National Health Commission of the People's Republic of China. The data request must

specify the research purpose, specific method, expected results, results sharing plan, and whether it involves ethics and other details. Expect a time frame of at least 6-8 months for data requests to be processed.

Field-specific reporting

Please select the one below that is the best fit for your research. If you are not sure, read the appropriate sections before making your selection.

☐ Life sciences ☐ Behavioural & social sciences ☒ Ecological, evolutionary & environmental sciences

For a reference copy of the document with all sections, see nature.com/documents/nr-reporting-summary-flat.pdf

Ecological, evolutionary & environmental sciences study design

All studies must disclose on these points even when the disclosure is negative.

Study description	Using a national representative data of 11,714,947 pregnant women in China, we explored the changes in the preterm birth rate during the COVID-19 lockdown period, and further explored the concurrent changes in the stillbirth rate using an interrupted-time-series analysis.
Research sample	Individual maternal data were collected from China s National Maternal Near Miss Surveillance System (NMNMSS). The NMNMSS collects the sociodemographic and obstetric information of pregnant and postpartum women from obstetric departments. The age range of participants was from 11 years to 60 years.
Sampling strategy	The sampling urban districts and rural counties of the National Maternal Near Miss Surveillance System (NMNMSS), which the surveillance health facilities located in, were based on China s National Maternal and Child Health Surveillance System (NMCHSS) and Provincial-level Maternal and Child Health Surveillance Systems (PMCHSSs). The surveillance sites of NMNMSS (326 urban districts and rural counties) were sampled randomly from combined NMCHSS and PMCHSSs within strata to ensure proportional representation of urban and rural populations across eastern, central, and western regions in China. Refer to the health facility selection criteria of WHO Global Survey for monitoring maternal and perinatal, once the surveillance sites are selected, two public health facilities located in these areas with more than 1000 deliveries per year are randomly selected. All the hospitals in Tibet were excluded due to the lack of skilled surveillance staff. Since all the analysis were based on the multi-variable models, and the number of independent variables was less than 15, the sample size of the study (more than 2 millions) were exceeds the requirements (over 10-fold of independent variables).
Data collection	In each sampled health facility, data collection began when a pregnant woman was hospitalized in obstetrics department, until she was discharged from hospital or left obstetric department. An adapted individual survey form was used to collected information on sociodemographic characteristics, pregnancy complications and terminations, interventions and process indicators, and maternal and perinatal outcomes. This individual survey form was modified according to the WHO Multicountry Survey on Maternal and Newborn Health. Data were collected by obstetricians or nurses responsible for patient care mainly through the medical records review. When the individual survey form was completed, data were entered onto a web-based online reporting system centralized at the National Office for Maternal and Child Health Surveillance of China (NOMCHS)
Timing and spatial scale	The spatial scale was set by the distribution site of surveillance hospital. The surveillance hospitals in NMNMSS are located in 326 districts or counties throughout 30 provinces in mainland China, excluding Tibet. The set of timing scale was based on following reasons: The Individual maternal data was based on delivery date of offsprings, which were from Jan 2012 to Dec 2020. We defined February 2020 as an important cut-off point in the present study, then divided the study period into two parts: baseline (January 1, 2012 to January 31, 2020) and the intervention stage (February 1, 2020 to December 31, 2020). As a result, 108 monthly preterm birth rates were included in the analysis.
Data exclusions	12,294,471 women delivered at least one baby who was more than or equal to 28 weeks of gestation or 1,000g or more birthweight during the study period. After excluding pregnancies lacking data, 11,714,947 were included in the final analysis (11,504,271 mothers of singletons and 210,676 of multiples). The exclusion criteria were pre-established.
Reproducibility	N/A. This study is an observational study
Randomization	N/A. This study is an observational study
Blinding	N/A. This study is an observational study

Did the study involve field work? ☐ Yes ☒ No

Reporting for specific materials, systems and methods

We require information from authors about some types of materials, experimental systems and methods used in many studies. Here, indicate whether each material, system or method listed is relevant to your study. If you are not sure if a list item applies to your research, read the appropriate section before selecting a response.

Materials & experimental systems

n/a	Involved in the study	
☒	☐	Antibodies
☒	☐	Eukaryotic cell lines
☒	☐	Palaeontology and archaeology
☒	☐	Animals and other organisms
☐	☒	Human research participants
☒	☐	Clinical data
☒	☐	Dual use research of concern

Methods

n/a	Involved in the study	
☒	☐	ChIP-seq
☒	☐	Flow cytometry
☒	☐	MRI-based neuroimaging

Human research participants

Policy information about studies involving human research participants

Population characteristics	The sociodemographic characteristics of the women who gave birth before and after COVID-19 lockdown measures were implemented are presented in Table 1. The proportion of singleton births among mothers with an advanced age (≥35 years) increased gradually from 9.1% to 12.0% during the study period, while multiple births increased from 11.3% to 13.8%. Moreover, the proportion of singleton and multiple pregnancies in the advantaged population increased slightly. However, the maternal characteristics were the same in the months before (9 months immediately prior to the intervention) and after COVID-19 lockdown measures were implemented.
Recruitment	All patients who delivered in the surveillance hospitals were recruited. The possible bias was that the National Maternal Near Miss Surveillance System may oversampled large referral hospitals in urban districts, due to the difference between the population covered by the surveillance system and the whole Chinese maternal population.
Ethics oversight	No patients were directly involved in the design, recruitment, development, or interpretation of the study. The NMNMSS data was approved by the ethics committee of the West China Second University Hospital, Sichuan University, China (Protocol ID: 2012008).

Note that full information on the approval of the study protocol must also be provided in the manuscript.
